# Changes in microbial communities, photosynthesis and calcification of the coral *Acropora gemmifera* in response to ocean acidification

**DOI:** 10.1038/srep35971

**Published:** 2016-10-27

**Authors:** Guowei Zhou, Tao Yuan, Lin Cai, Weipeng Zhang, Renmao Tian, Haoya Tong, Lei Jiang, Xiangcheng Yuan, Sheng Liu, Peiyuan Qian, Hui Huang

**Affiliations:** 1 Key Laboratory of Tropical Marine Bio-resources and Ecology, South China Sea Institute of Oceanology, Chinese Academy of Sciences, China; 2 Tropical Marine Biological Research Station in Hainan, Chinese Academy of Sciences, China; 3 Shenzhen Research Institute and Division of Life Science, Hong Kong University of Science and Technology, Hong Kong SAR, China

## Abstract

With the increasing anthropogenic CO_2_ concentration, ocean acidification (OA) can have dramatic effects on coral reefs. However, the effects of OA on coral physiology and the associated microbes remain largely unknown. In the present study, reef-building coral *Acropora gemmifera* collected from a reef flat with highly fluctuating environmental condition in the South China Sea were exposed to three levels of partial pressure of carbon dioxide (*p*CO_2_) (i.e., 421, 923, and 2070 μatm) for four weeks. The microbial community structures associated with *A. gemmifera* under these treatments were analyzed using 16S rRNA gene barcode sequencing. The results revealed that the microbial community associated with *A. gemmifera* was highly diverse at the genus level and dominated by Alphaproteobacteria. More importantly, the microbial community structure remained rather stable under different *p*CO_2_ treatments. Photosynthesis and calcification in *A. gemmifera*, as indicated by enrichment of δ^18^O and increased depletion of δ^13^C in the coral skeleton, were significantly impaired only at the high *p*CO_2_ (2070 μatm). These results suggest that *A. gemmifera* can maintain a high degree of stable microbial communities despite of significant physiological changes in response to extremely high *p*CO_2_.

Rising CO_2_ in the atmosphere elevates the partial pressure of carbon dioxide (*p*CO_2_) in seawater and reduces the global oceanic pH and carbonate ion concentrations, which is called ocean acidification (OA). It has been suggested that OA has profound effects on marine organisms and ecosystems, particularly calcifying organisms such as reef-building corals^1^,^2^,^3^. With the increasing OA associated with ocean warming due to the rising global CO_2_ emissions^2^, there is an urgent need to understand and predict the tolerance and response of corals to future climate change.

Reef-building corals are commonly referred to as holobionts, which comprise coral host and associated microorganisms including endosymbiotic photosynthetic algae, bacteria, and archaea, among others. These complex microbial partners play pivotal roles in coral health and holobiont function in carbon, nitrogen and sulfur cycles. The future fate of coral reefs largely depends on the capacity of corals and their symbionts to acclimatize or adapt to climate change^2^,^4^,^5^,^6^,^7^. There is emerging evidence that corals can adapt to climate change^8^, although coral photosynthesis and growth can be negatively impacted by OA^9^. Moreover, flexible coral-algal symbiosis may facilitate the acclimatization/adaptation of the holobiont through algal shuffling or switching^10^,^11^.

Shifting in the composition of coral-associated microbiota has been observed following environmental disturbances (e.g., elevated temperature) and has often been linked to impaired host health^12^,^13^,^14^, although it has also been hypothesized to mediate holobiont resistance to environmental perturbations^6^,^15^. Coral-associated microbial communities may be affected directly or indirectly by OA and would subsequently compromise holobiont fitness (i.e., changes in photosynthesis or calcification) and survival, possibly due to a shift in the functional roles of microbial associations^12^,^16^,^17^,^18^. However, to date it is not clear how microbial communities change in coral in response to OA, or whether theses changes also alter host physiology. Preliminary laboratory-based investigations have revealed a remarkable impact of increased *p*CO_2_ or reduced pH on coral microbial communities^12^,^16^,^18^. In contrast, no significant changes were observed in the microbial communities of transplanted corals in natural CO_2_ vents^19^ and associated with two Pacific corals after 8 weeks of exposure to increased *p*CO_2_^20^. These contradictory findings underscore the need for further research. Most recently, microbial communities associated with coral and sponge from natural CO_2_ seeps have demonstrated species-specific acclimatization to their habitats^21^. Therefore, the potential of natural microbial communities in corals to acclimatize/adapt to OA cannot be overlooked.

Natural fluctuations in seawater pH/*p*CO_2_ are common, especially diel pH/*p*CO_2_ fluctuations in shallow water coral reefs^22^ and these fluctuations affect the abundance and distribution of marine organisms^23^. The fauna have been suggested to be locally acclimatized/adapted to the variable pH environment as an evolutionary mechanism to cope with future acidification^24^,^25^,^26^. However, the adaptation of the coral holobionts to OA remains largely unexplored and is worth careful investigation.

The Luhuitou fringing reef (18°12′N, 109°28′E) is located in the southern Hainan Island, South China Sea (see Supplementary Fig. S1) and used to have a high coverage of living coral, which has declined by 80% since the 1960s^27^. The diurnal and seasonal variation of the reef flat seawater pH/*p*CO_2_ is high^28^,^29^,^30^, and the recorded extreme level is even lower than the value currently predicted at the end of this century (see Supplementary Table S1). The rapidly-growing branching coral *Acropora*, which is distributed worldwide, is an ecologically important genus in this reef flat. In the present study, *A. gemmifera* colonies collected from this reef flat were exposed to three *p*CO_2_ levels to test our hypothesis that both the coral physiology and the microbial communities associated with this coral species are stable and resistant to OA exposure.

## Results and Discussion

### Overview of the microbial communities

After quality filtering, 308,591 reads were used for the downstream analyses. The number of operational taxonomic units (OTUs), Chao1 estimation of species richness, and Shannon index were obtained at a dissimilarity of 3% (Table 1). The rarefaction analyses revealed that the sequencing effort for each sample was sufficient to reflect the microbial diversity, and the rank-abundance curve showed that most OTUs had an abundance lower than 0.1%, which demonstrated that the microbial communities were occupied by rare species (see Supplementary Fig. S2). The prevalence of rare species has been widely demonstrated elsewhere, yet the ecological and functional roles of these rare species remain unknown^31^. There were no significant differences in beta diversity among the *p*CO_2_ treatments, which is in contrast to the findings of a previous report showing an increase in coral microbial diversity with decreasing pH, possibly caused by an intermediate disturbance^16^.

**Figure 1:**
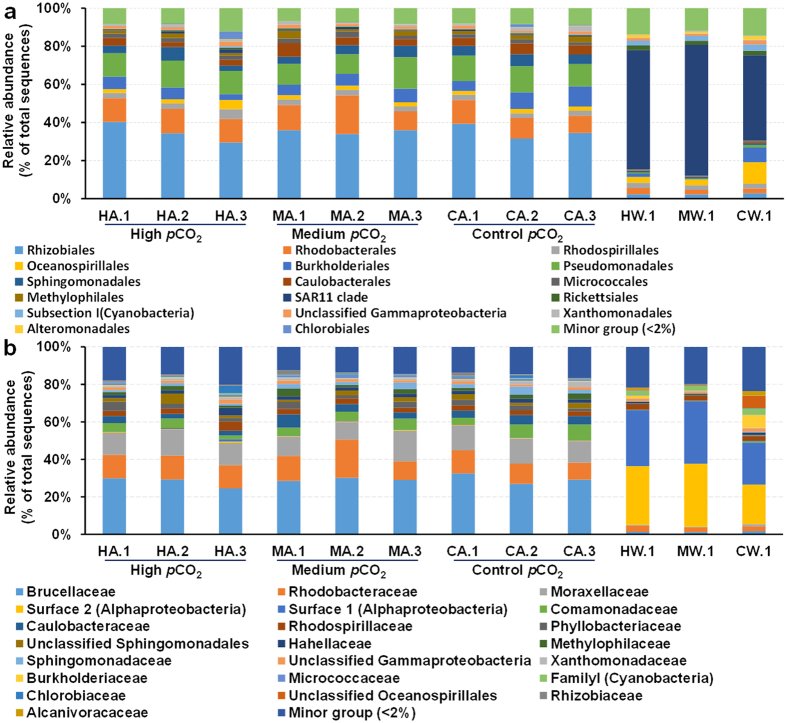
Coral and seawater microbial communities at the order (**a**) and family (**b**) level. The minor group represents the sum of all orders or families representing <2% in all samples. “H”, “M” and “C” refer to high, medium and control *p*CO_2_ treatment, respectively. “A” and “W” refer to coral and seawater samples, respectively. The number following the letter indicates a replicate.

In total, 24 bacterial and 2 archaeal phyla were detected in the coral and seawater samples, including Proteobacteria, Actinobacteria, Bacteroidetes, Cyanobacteria, Firmicutes, Thaumarchaeota and Euryarchaeota (see Supplementary Fig. S3). The relative abundance of archaea made up less than 0.1% of the seawater samples, with most OTUs belonging to autotrophic ammonia-oxidizing archaea (AOA) within the phylum Thaumarchaeota, which was even less abundant in the coral samples. It has been suggested that archaea may play prominent roles in corals and reefs^5^, although their abundance in both corals and reef water is much lower than that of bacteria^32^. Notably, the bacterial communities in both *A. gemmifera* and seawater were dominated by Proteobacteria and the most abundant class was Alphaproteobacteria (56–80%), among which the majority were assigned to the family Brucellaceae in the order Rhizobiales (Fig. 1) followed by Gammaproteobacteria (9–26%). Both Alphaproteobacteria and Gammaproteobacteria are commonly highly abundant in corals, but their relative abundance varies among species^33^.

**Figure 2:**
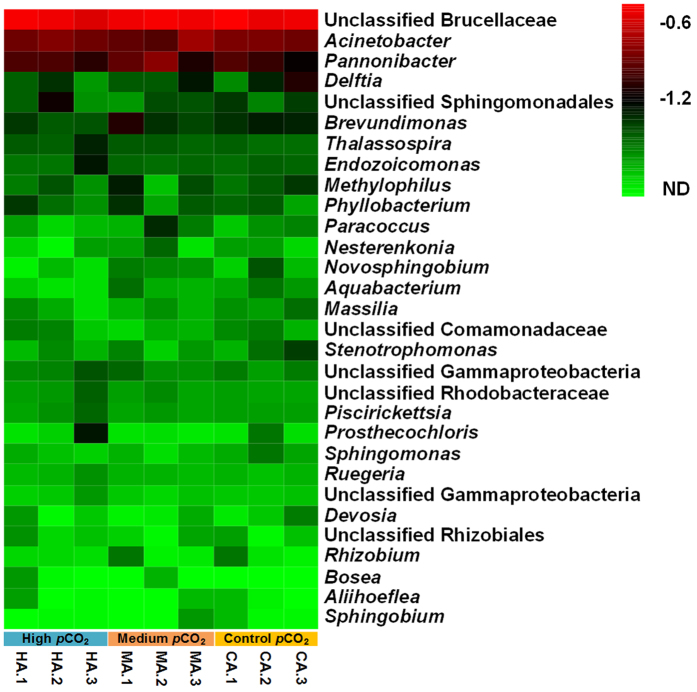
Heat map showing the abundance of microbial assignments in coral at the genus level. Genera abundance >1% at least in one sample are shown. The abundance values are log_10_(x + 0.01)-transformed for plotting. For the heat map scale, “ND, −1.2, and −0.6” indicate relative abundance “0, 5%, and 24%”, respectively. The heat map was generated with R (version 3.1.3, R Development Core Team, 2015). “H”, “M” and “C” refer to high, medium and control *p*CO_2_ treatment, respectively. “A” refer to coral samples. The number following the letter indicates a replicate.

Taxonomic assignment at the genus level was summarized, and genera with an abundance of greater than 1% in at least one sample are shown in a heat map (Fig. 2). In the present study, the unclassified Brucellaceae (>24%), *Acinetobacter* (>9%) and *Pannonibacter* (>5%) were the most abundant genera in coral, regardless of the *p*CO_2_ treatment. Diazotrophs within the order Rhizobiales have been found in other coral species and were considered to be important for coral holobiont in nitrogen-limited waters^5^. It has been shown that copiotrophic taxa including Brucellaceae were enriched in algal-dominated environment^34^. Diverse algal communities on the Luhuitou fringing reef^35^ might contribute to the dominance of unclassified Brucellaceae in *A. gemmifera*. *Acinetobacter* spp. have also been commonly reported in bleached and healthy corals^36^. Therefore, it is reasonable to suggest that the dominant genera, including *Acinetobacter* and the unclassified Brucellaceae, play critical roles in *A. gemmifera*. Interestingly, the putatively endosymbiotic *Endozoicomonas*^37^ was detected at a very low level in all coral samples (<2%). The photosynthetic Cyanobacteria assigned to the genus *Synechococcus* have been reported in sponge and coral^21^ and were also detected at a very low abundance (0.2%) in *A. gemmifera*. However, the functions of bacteria and archaea and their interactions in the coral holobiont remain largely unclear.

### Stability of microbial communities in *A. gemmifera*

As estimated by Adonis analysis at all taxonomic levels (Adonis test, *p* > 0.05) and nMDS ordination (see Supplementary Fig. S4), there were no significant differences in microbial community compositions in *A. gemmifera* among the different *p*CO_2_ treatments even after a 4-week exposure. Additionally, results from the SIMPER analysis showed that the dissimilarity of microbial communities among *p*CO_2_ treatments was very small (see Supplementary Fig. S5). Taken together, these findings suggest that the *A. gemmifera* microbiome was not significantly affected by elevated *p*CO_2_ and could remain relatively stable (Fig. 2). This result is inconsistent with the findings of some previous studies in which the coral microbiome shifted under higher *p*CO_2_ or lower pH treatments over treatment periods ranging from days to months^16^,^38^. However, our finding is consistent with some other studies. For example, there were no differences in the microbial community structure in coral between pH 7.7 (*p*CO_2_ = 1187 μatm) and 7.5 (*p*CO_2_ = 1638 μatm) whereas a significant difference was observed between pH 8.1 (*p*CO_2_ = 464 μatm) and 7.9 (*p*CO_2_ = 822 μatm) after 6 weeks of CO_2_ exposure^18^. Moreover, Meron *et al*.^19^ observed no significant changes in microbial communities associated with two Mediterranean coral species that were transplanted along natural pH gradients. A recent study reported that the microbial communities of two Pacific coral species were tolerant to reduced pH 7.9 (*p*CO_2_ 738–835 μatm)^20^. These inconsistent results might reflect that some coral-microbial associations are more resistant to increases in *p*CO_2_/decreases in pH than others, but these findings could also be partially attributed to differences in the experimental conditions (e.g., field *vs* laboratory, *p*CO_2_ or pH level, among others), and the exposure duration.

In most cases, microbial communities are dynamic and can rapidly respond to OA in seawater^39^, biofilms^40^ and other associated systems^41^. The genus *Acropora* is among the most sensitive coral to environmental change^42^. The potential for coral acclimatization or adaptation to climate change has been studied^43^, and the physiological and molecular mechanisms responsible for OA resistance have recently been proposed^8^. Although there is limited evidence for biological adaptation to climate change in coral microbial symbionts, the adaptive power to climate change has been well documented in the photosymbiotic *Symbiodinium*^10^,^11^,^44^. The shallow habitat of the coral *A. gemmifera* sampled in the present study has been experiencing regular large diurnal and seasonal variations in pH/*p*CO_2_ (see Supplementary Table S1), which are mainly driven by biological activities of the reef^28^,^29^,^30^. Therefore, it is most likely that microbial communities harbored by the natural population of *A. gemmifera* are resistant to the increased *p*CO_2_, due to long-term acclimatization/adaptation to the highly dynamic pH conditions within the reef flat. Thus, there may be a resilient relationship between coral and microbial partners that can help corals overcome the fluctuations in seawater pH/*p*CO_2_. However, we note that the stability of the coral microbiome is based on only one species colleting from a fluctuating environment. The application of variable *p*CO_2_ conditions and controls from stable pH/*p*CO_2_ environments in highly replicated culture experiments with consideration of tank effects could further confirm this assumption in future studies.

A recent study supports this interpretation. Morrow *et al*.^21^ found that microbial communities associated with coral and sponge originally from natural volcanic CO_2_ seeps were distinct from the nearby control sites, reflecting the acclimatization of the host-symbiont to the high *p*CO_2_ environment. Local acclimatization/adaptation to environmental variations in *p*CO_2_, temperature and nutrients, among others, has revealed the capabilities of marine organisms including reef-building corals and symbiotic algae, to adapt to future climate change^8^,^24^,^25^,^44^,^45^. However, in general, the species-specific response of marine organisms to OA remains poorly understood^1^,^23^,^46^. Thus, it is rather premature to conclude whether we can extrapolate the adaptive power of coral and its associated microbes documented in the present study to other coral species living in highly fluctuating reef environments.

### Skeletal isotopic response to ocean acidification

**Figure 3:**
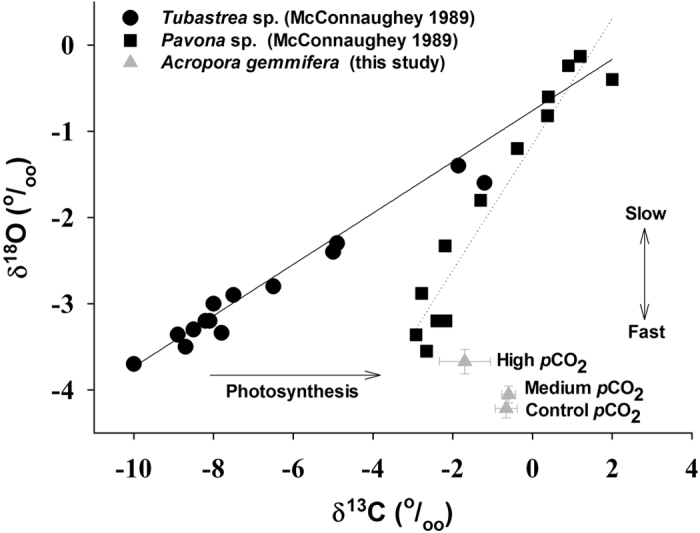
Skeletal isotopic response of *Acropora gemmifera* cultured under the control (421 μatm) and increased (923 μatm and 2070 μatm) *p*CO_2_ conditions. Skeletal isotopic composition of non-photosynthetic coral *Tubastrea* sp. and photosynthetic coral *Pavona* sp. reported in a previous study (McConnaughey 1989) are plotted for comparison. Photosynthesis indicated the carbon isotopic offset due to respiration and photosynthesis. “Slow” and “Fast” indicated slow and fast coral calcification rates. Fast growing *Acropora* corals are expected to have more enriched δ^13^C but depleted δ^18^O values.

During our experiments, all blocks of *A. gemmifera* exposed to the different *p*CO_2_ treatments grew, survived and formed new skeleton (see Supplementary Fig. S6), even at the high *p*CO_2_ (pH reduced to 7.47). When the fast-growing coral *A. gemmifera* skeletal δ^13^C and δ^18^O were compared among the different *p*CO_2_ treatments, the skeletal δ^13^C values in *A. gemmifera* were significantly different between any two *p*CO_2_ treatments except between the control and the medium (Fig. 3). Skeletal δ^13^C values were depleted by 1.10‰ and 1.04‰ for the control *vs.* high *p*CO_2_ and for the medium *vs.* high *p*CO_2_, respectively (one-way ANOVA, Tukey test, *p* < 0.05). Skeletal δ^18^O values in *A. gemmifera* were enriched with increased *p*CO_2_; they were 0.55‰ and 0.38‰ heavier in response to high *p*CO_2_ than those in the control and medium, respectively (one-way ANOVA, Tukey test, *p* < 0.05). Compared with the previous data^47^, skeletal δ^18^O values revealed greater depletion in fast-growing coral *A. gemmifera*, while the skeletal δ^13^C values remained within range. In addition, the relationship between skeletal δ^13^C and δ^18^O in the non-photosynthetic coral *Tubastrea* sp. deviated the most from those both in the photosynthetic corals *A. gemmifera* and *Pavona* sp. (Fig. 3).

The isotopic composition of the coral skeleton can be affected by metabolic isotope effects (e.g., photosynthesis and respiration) and kinetic isotope effects (e.g., the calcification process)^48^. The coral skeletal δ^13^C and δ^18^O were generally used as an effective proxy to study photosynthesis, respiration and calcification processes^48^,^49^. In general, photosynthesis and calcification can enrich skeletal δ^13^C but deplete skeletal δ^18^O due to isotope fractionation^47^,^49^. Compared non-photosynthetic (*Tubastrea* sp.) and photosynthetic (*Pavona* sp.) corals^47^, the relationship of δ^13^C *vs.* δ^18^O in *Pavona* sp. and *A. gemmifera* was different from that in *Tubastrea* sp. (non-photosynthetic coral) due to active photosynthesis. In addition, more δ^18^O deviation was observed in *A. gemmifera* than *Tubastrea* sp. and *Pavona* sp., mostly due to the highest growth rate in *A. gemmifera* (Fig. 3). Skeletal δ^13^C values in *A. gemmifera* were significantly depleted at the high *p*CO_2_, suggesting that the photosynthetic rates were much lower at the high *p*CO_2_ than at the control *p*CO_2_. The variation in skeletal δ^18^O values of *A. gemmifera* was consistent with the findings of a previous study demonstrating an enrichment of δ^18^O in the coral skeleton in response to elevated *p*CO_2_^49^. The coral calcification rate decreases under reduced pH conditions^9^, which corresponds to heavier skeletal δ^18^O, whereas low *p*CO_2_ and higher pH lead to species with lighter δ^18^O because HCO_3_^−^ is isotopically heavier than CO_3_^2^,^3^,^4^,^5^,^6^,^7^,^8^,^9^,^10^,^11^,^12^,^13^,^14^,^15^,^16^,^17^,^18^,^19^,^20^,^21^,^22^,^23^,^24^,^25^,^26^,^27^,^28^,^29^,^30^,^31^,^32^,^33^,^34^,^35^,^36^,^37^,^38^,^39^,^40^,^41^,^42^,^43^,^44^,^45^,^46^,^47^,^48^,^49^. Consequently, the significantly enriched δ^18^O and more depleted δ^13^C in *A. gemmifera* observed herein may reflect slight reductions in photosynthesis and calcification at the high *p*CO_2_. It should be noted that *A. gemmifera* skeletal δ^13^C and δ^18^O values did not vary significantly at the medium *p*CO_2_, potentially because this stress level did not exceed its acclimatization range. These findings indicate that the coral *A. gemmifera* is able to acclimate to an acidifying ocean, even in the presence of a dramatically increasing atmospheric CO_2_ concentration.

Although the mechanisms by which extremely high *p*CO_2_ induces decreased photosynthesis and calcification efficiencies in *A. gemmifera* are unknown, several potential mechanisms have been proposed, such as photoinhibition and suppression of the carbon concentrating process^3^,^9^. Photosynthesis, calcification and other physiological processes in reef-building corals can be influenced by their microbial partners or vice versa under OA^17^. However, the microbial communities associated with *A. gemmifera* remained unchanged as a consequence of host physiological changes, further supporting our hypothesis that highly stable microbial associations are likely driven by local acclimatization/adaptation to the fluctuating environment. Alternatively, host physiological costs might result from a potentially increasing energy demand to maintain stable microbial assemblages at the extremely high *p*CO_2_ that exceeds its tolerance level.

It has also been proposed that physiological differences among symbiotic algal phylotypes may influence the stable isotopic composition of coral skeleton^50^. Furthermore, the distinct mechanisms used to concentrate carbon by different *Symbiodinium* phylotypes and their physiological responses to OA are phylotype- specific^51^. For example, *Symbiodnium* community shifts may occur in response to environmental stresses^10^,^11^. In the present study, we did not investigate *Symbiodinium* phylotypes associated with *A. gemmifera*. A recent study found no changes in *Symbiodinium* phylotypes associated with corals among different pH conditions^19^, suggesting the presence of stable *Symbiodinium* assemblages in corals in response to OA. In general, a stable microbial partnership to maintain key metabolic functions can improve coral holobiont acclimatization or adaptation to environmental stresses^5^. However, the interactions between microbial communities and coral physiology remain far from clarified.

## Conclusions

In this study, the tropical fast-growing coral *A. gemmifera* from a shallow habitat with natural pH/*p*CO_2_ fluctuations was selected as a representative species and was exposed to a 4-week CO_2_ treatment. The microbial communities and skeletal isotopic compositions were examined simultaneously. We found that the microbial communities in *A. gemmifera* remained remarkably stable. In contrast, neither photosynthesis nor calcification in the coral were impacted under medium *p*CO_2_ but were both negatively affected under extremely high *p*CO_2_, as demonstrated by an enrichment of δ^18^O and increased depletion of δ^13^C in the skeleton under extremely high CO_2_ stress. The present findings indicate that some reef-building corals may be more tolerant to OA in pH/*p*CO_2_ fluctuating environments and have a high degree of host-symbiont fidelity, despite the observed impairment of host physiological processes in response to high CO_2_ stress. This study also contributes to our understanding of the variability of OA resistance among coral-microbial associations. Because coral reefs are facing other environmental stresses in addition to OA, the synergistic effects of multiple stressors on the coral microbiome must be carefully examined to understand the persistence of the coral holobiont and coral reefs in the future ocean.

## Methods

### Experimental design and sample collection

The experiment was conducted in an outdoor seawater flow-through system at the Tropical Marine Biological Research Station in Hainan (TMBRS) near the Luhuitou fringing reef. Seawater was pumped directly from a depth of 6 m in the front of the TMBRS into three 2000-L header tanks in which the designated *p*CO_2_ level was adjusted with CO_2_ gas. Three *p*CO_2_ treatments were projected current *p*CO_2_ levels, those at the end of the present century, and double the end of the present century: 421 μatm, 923 μatm, and 2070 μatm with pH values of 8.07, 7.76 and 7.47, respectively. The well-mixed seawater from the header tank continually flowed into three aquaria at a rate of 0.5 l min^−1^. Each aquaria was equipped with a submerged pump to drive the water flow, and all aquaria were maintained under a natural light–dark cycle to mimic the field condition.

Six healthy colonies of *A. gemmifera* were collected from the Luhuitou fringing reef flat at a depth of ~2 m in May 2014 and divided into small pieces. After acclimation for two weeks in large aquaria with running water, one coral nubbin was randomly selected and was suspended using fine nylon strings in three aquaria for exposure to each of the three *p*CO_2_ treatments. The coral nubbins from same colony were evenly distributed among the *p*CO_2_ treatments to avoid any possible sampling bias. A total of 9 coral nubbins were maintained for further analysis during the experiment.

At the end of the experiment (i.e., 4-week exposure), 1 L of seawater from each treatment was filtered through 0.2 μm polycarbonate (PC) membrane filters and stored at −20 °C for further analyses. One coral nubbin from each treatment aquarium was sampled and divided into two aliquots. One was rinsed three times and then preserved in 70% ethanol and stored at −20 °C for DNA extraction; the other was used for stable isotope analyses.

### Determination of environmental parameters

Photosynthetically active radiation (PAR) in each aquarium was recorded every 30 min below the seawater surface using the Hobo^®^ logger (Onset, USA). The average diurnal variations in PAR during the 4-week period are shown in Fig. S7. The seawater pH and temperature were measured daily in each aquarium using a pH meter (Orion Star™) and the total alkalinity was also determined weekly using an automated titration system (Metrohm 877 Titrino plus, Switzerland). Carbonate system parameters, including [HCO_3_^−^], [CO_3_^2−^], *p*CO_2_ and aragonite saturation state (Ω_A_), were calculated from the measured pH and total alkalinity values using the CO2SYS program^52^ (Table 2).

### DNA extraction and 16S rDNA amplicon sequencing

A preserved piece of coral was homogenized in liquid nitrogen and then the total DNA from the resulting coral powder and filtered seawater samples was subsequently extracted using the Fast DNA^®^ SPIN Kit for Soil (MP Biomedicals, Irvine, CA) according to the company’s protocol. The DNA samples were amplified by PCR using barcoded primers targeting the hypervariable region V3-V4 of the 16S rRNA gene of Bacteria and Archaea: 341F (5′-CCTAYGGGRBGCASCAG-3′) and 802R (5′-TACNVGGGTATCTAATCC-3′)^53^. The PCR amplification was performed using a thermocycle controller (MJ Research Inc., Bio-Rad) with the following program: an initial denaturation at 94 °C for 5 min, followed by 35 cycles at 94 °C for 30 s, 50 °C for 30 s, 72 °C for 30 s, and a final extension at 72 °C for 5 min. All PCR products were purified using the Qiagen Agarose Gel DNA Purification Kit (Qiagen, Germany) and quantified using a NanoDrop device (Thermo Scientific, USA). All amplicon products were mixed at equal concentrations and sequenced on an Illumina Miseq platform using 2 × 300 bp mode at Novogene (Beijing, China). The raw reads were submitted to the NCBI Sequence Read Archive under accession number SRP066229 (SRR2917919).

### Sequence data processing

Overlapping paired-end reads were merged to obtain full-length 16S V3-V4 fragments using PEAR^54^. After de-multiplexing and quality control, the downstream bioinformatics analysis was performed with QIIME1.5.0 pipelines^55^. Briefly, OTUs with 97% similarity were defined after the qualified reads were clustered using Uclust^56^. Representative sequences for each OTU were assigned to different taxa using the Ribosomal Database Project (RDP) classifier version 2.2^57^ against the SILVA108 database^58^ with a 50% cut-off threshold. Representatives assigned to eukaryotes, chloroplasts and mitochondria were filtered out. The taxon and abundance were summarized at the phylum, class, order, family, and genus levels. The species diversity, Shannon index, rarefaction curves and rank-abundance curves were determined using the QIIME pipeline.

### Stable isotope analyses

Skeleton fragments of *A. gemmifera* were soaked in 30% hydrogen peroxide to remove coral tissue and then sonicated for 4 min at 20 °C. Skeletons were subsequently washed several times with double-distilled water and dried overnight at 50 °C. The newly grown part was scalped and ground into powder. Coral skeletal δ^13^C and δ^18^O data were obtained using a Finnigan MAT 253 Isotope Ratio Mass Spectrometer coupled to a Kiel Carbonate Device IV at the South China Sea Institute of Oceanology, Chinese Academy of Sciences, China. δ^13^C and δ^18^O were determined by repeated measurements of the international reference standard NBS-18. δ^13^C and δ^18^O values were presented as the per mil (‰) deviation of ^13^C/^12^C and ^18^O/^16^O, respectively.

### Statistical analyses

To test the effect of *p*CO_2_ treatments on microbial community compositions of coral samples, after normalizing all sequence reads for taxonomic analysis to the lowest sequencing depth (19,098 reads), pairwise dissimilarities among coral samples were calculated based on the Bray–Curtis index for ‘Adonis’, which is a non-parametric multivariate analysis of variance. In the Adonis analysis, the distance matrix was the response variable with *p*CO_2_ treatment as independent variable. Non-metric multidimensional scaling (nMDS) was also performed to visualize the dissimilarities. Genera making the greatest contribution to dissimilarity among the *p*CO_2_ treatments were further investigated through similarity percentage (SIMPER) analysis. To compare coral skeletal δ^13^C and δ^18^O among *p*CO_2_ treatments, one-way ANOVA and Tukey’s test were employed. All analyses were conducted using the package vegan within the R statistical environment (version 3.1.3, R Development Core Team, 2015).

## Additional Information

**How to cite this article**: Zhou, G. *et al*. Changes in microbial communities, photosynthesis and calcification of the coral *Acropora gemmifera* holobiont in response to ocean acidification. *Sci. Rep.* **6**, 35971; doi: 10.1038/srep35971 (2016).

**Publisher’s note:** Springer Nature remains neutral with regard to jurisdictional claims in published maps and institutional affiliations.

## Supplementary Material

Supplementary Information

## Figures and Tables

**Table 1:** Sample information and summary of microbial communities in corals and seawaters.

Sample ID	Treatment	Number of qualified reads	Total OTUs	Chao1 Ave.	Observed species Ave.	Shannon Ave.
HA.1	High *p*CO_2_ treatment	37600	1301	1678.248	707.2	5.420
HA.2		19098	1115	1826.916	874.8	5.494
HA.3		29468	1687	2102.160	1087	6.333
MA.1	Medium *p*CO_2_ treatment	27489	1108	1545.223	707.2	5.360
MA.2		27946	1295	1780.889	822.9	5.316
MA.3		26650	1074	1528.017	709.3	5.431
CA.1	Control *p*CO_2_ treatment	24616	1065	1629.961	716.8	5.258
CA.2		21224	989	1576.258	727.8	5.527
CA.3		28163	1244	1688.842	783.5	5.450
HW.1	High *p*CO_2_ treatment	14358	1237	1901.201	1152.6	5.252
MW.1	Medium *p*CO_2_ treatment	15688	1203	1795.261	1075.2	4.860
CW.1	Control *p*CO_2_ treatment	12463	1127	1886.261	1126.7	5.908

“H”, “M” and “C” refer to high, medium and control *p*CO_2_ treatment, respectively. “A” and “W” refer to coral and seawater samples, respectively. The number following the letter indicates a replicate. Chao1, Observed species and Shannon index were determined at 3% dissimilarity after normalizing the full 16S dataset (including bacterial and archaeal sequences) to 12,459 sequences per sample.

**Table 2:** Carbonate chemistry parameters of seawater for each treatment.

Treatment	pH_NBS_	Alkalinity (μmol kg^−1^)	HCO_3_^−^ (μmol kg^−1^)	CO_3_^2−^ (μmol kg^−1^)	*p*CO_2_ (μatm)	Ωara
Control *p*CO_2_	8.07 ± 0.02	2233 ± 22	1701 ± 35	214 ± 15	421 ± 49	3.6 ± 0.25
Medium *p*CO_2_	7.76 ± 0.02	2223 ± 11	1912 ± 36	125 ± 11	923 ± 113	2.1 ± 0.19
High *p*CO_2_	7.47 ± 0.02	2230 ± 13	2089 ± 25	67 ± 6	2070 ± 259	1.1 ± 0.01

Values are means ± SE. Seawater pH (NBS scale), and salinity (31.5) were measured daily during the experiment (n = 28). Total alkalinity (TA) was measured at a specific time point every week (n = 4). The remaining parameters for carbonate seawater chemistry were calculated using the CO_2_SYS program.

## References

[b1] Kroeker K. J., Kordas R. L., Crim R. N. & Singh G. G. Meta-analysis reveals negative yet variable effects of ocean acidification on marine organisms. Ecol Lett 13, 1419–1434, 10.1111/j.1461-0248.2010.01518.x (2010). 20958904

[b2] Pandolfi J. M., Connolly S. R., Marshall D. J. & Cohen A. L. Projecting coral reef futures under global warming and ocean acidification. Science 333, 418–422, 10.1126/science.1204794 (2011). 21778392

[b3] Hofmann G. E. . The effect of ocean acidification on calcifying organisms in marine ecosystems: an organism-to-ecosystem perspective. Annu Rev Ecol Evol S, 41, 127–147, 10.1146/annurev.ecolsys.110308.120227 (2010).

[b4] Ainsworth T. D., Thurber R. V. & Gates R. D. The future of coral reefs: a microbial perspective. Trends Ecol Evol 25, 233–240, 10.1016/j.tree.2009.11.001 (2010). 20006405

[b5] Krediet C. J., Ritchie K. B., Paul V. J. & Teplitski M. Coral-associated micro-organisms and their roles in promoting coral health and thwarting diseases. P Roy Soc B-Biol Sci 280, 20122328, 10.1098/rspb.2012.2328 (2013). PMC3574386 23363627

[b6] Rosenberg E., Koren O., Reshef L., Efrony R. & Zilber-Rosenberg I. The role of microorganisms in coral health, disease and evolution. Nat Rev Microbiol 5, 355–362, 10.1038/nrmicro1635 (2007). 17384666

[b7] Rädecker N., Pogoreutz C., Voolstra C. R., Wiedenmann J. & Wild C. Nitrogen cycling in corals: the key to understanding holobiont functioning? Trends Microbiol 23, 490–497, 10.1016/j.tim.2015.03.008 (2015). 25868684

[b8] Palumbi S. R., Barshis D. J., Traylor-Knowles N. & Bay R. A. Mechanisms of reef coral resistance to future climate change. Science 344, 895–898, 10.1126/science.1251336 (2014). 24762535

[b9] Anthony K. R., Kline D. I., Diaz-Pulido G., Dove S. & Hoegh-Guldberg O. Ocean acidification causes bleaching and productivity loss in coral reef builders. Proc Natl Acad Sci USA 105, 17442–17446, 10.1073/pnas.0804478105 (2008). 18988740 PMC2580748

[b10] Berkelmans R. & van Oppen M. J. The role of zooxanthellae in the thermal tolerance of corals: a ‘nugget of hope’ for coral reefs in an era of climate change. P Roy Soc B-Biol Sci 273, 2305–2312, 10.1098/rspb.2006.3567 (2006). PMC1636081 16928632

[b11] LaJeunesse T. C. . Host-symbiont recombination versus natural selection in the response of coral-dinoflagellate symbioses to environmental disturbance. P Roy Soc B-Biol Sci 277, 2925–2934, 10.1098/rspb.2010.0385 (2010). PMC2982020 20444713

[b12] Thurber R. V. . Metagenomic analysis of stressed coral holobionts. Environ Microbiol 11, 2148–2163, 10.1111/j.1462-2920.2009.01935.x (2009). 19397678

[b13] Bourne D., Iida Y., Uthicke S. & Smith-Keune C. Changes in coral-associated microbial communities during a bleaching event. ISME J 2, 350–363, 10.1038/ismej.2007.112 (2008). 18059490

[b14] Roder C. . Bacterial profiling of White Plague Disease in a comparative coral species framework. ISME J 8, 31–39, 10.1038/ismej.2013.127 (2014). 23924783 PMC3869008

[b15] Reshef L., Koren O., Loya Y., Zilber-Rosenberg I. & Rosenberg E. The coral probiotic hypothesis. Environ Microbiol 8, 2068–2073, 10.1111/j.1462-2920.2006.01148.x (2006). 17107548

[b16] Meron D. . The impact of reduced pH on the microbial community of the coral *Acropora eurystoma*. ISME J 5, 51–60, 10.1038/ismej.2010.102 (2011). 20668489 PMC3105665

[b17] O’Brien P. A., Morrow K. M., Willis B. & Bourne D. Implications of ocean acidification for marine microorganisms from the free-living to the host-associated. Front Mar Sci 3, 47, 10.3389/fmars.2016.00047(2016).

[b18] Webster N. S. . Near-future ocean acidification causes differences in microbial associations within diverse coral reef taxa. Environ Microbiol Rep 5, 243–251, 10.1111/1758-2229.12006 (2013). 23584968

[b19] Meron D. . Changes in coral microbial communities in response to a natural pH gradient. ISME J 6, 1775–1785, 10.1038/ismej.2012.19 (2012). 22437157 PMC3498918

[b20] Webster N. . Host-associated coral reef microbes respond to the cumulative pressures of ocean warming and ocean acidification. Sci Rep 6, 19324, 10.1038/srep19324 (2016). 26758800 PMC4725835

[b21] Morrow K. M. . Natural volcanic CO_2_ seeps reveal future trajectories for host–microbial associations in corals and sponges. The ISME journal 9, 894–908, 10.1038/ismej.2014.188 (2015). 25325380 PMC4817704

[b22] Hofmann G. E. . High-frequency dynamics of ocean pH: a multi-ecosystem comparison. PLoS One 6, e28983, 10.1371/journal.pone.0028983 (2011). 22205986 PMC3242773

[b23] Fabricius K. E. . Losers and winners in coral reefs acclimatized to elevated carbon dioxide concentrations. Nat Clim Change 1, 165–169, 10.1038/nclimate1122 (2011).

[b24] Sunday J. M. . Evolution in an acidifying ocean. Trends Ecol Evol 29, 117–125, 10.1016/j.tree.2013.11.001 (2014). 24355315

[b25] Sanford E. & Kelly M. W. Local adaptation in marine invertebrates. Ann Rev Mar Sci 3, 509–535, 10.1146/annurev-marine-120709-142756 (2011). 21329215

[b26] Kelly M. W., Padilla-Gamino J. L. & Hofmann G. E. Natural variation and the capacity to adapt to ocean acidification in the keystone sea urchin *Strongylocentrotus purpuratus*. *Glob Chang* Biol 19, 2536–2546, 10.1111/gcb.12251 (2013). 23661315

[b27] Hughes T. P., Huang H. & Young M. A. The wicked problem of China’s disappearing coral reefs. Conserv Biol 27, 261–269, 10.1111/j.1523-1739.2012.01957.x (2013). 23140101

[b28] Zhang C. L. . Diurnal and seasonal variations of carbonate system parameters on Luhuitou fringing reef, Sanya Bay, Hainan Island, South China Sea. Deep-Sea Res Pt II 96, 65–74, 10.1016/j.dsr2.2013.02.013 (2013).

[b29] Chen X. F. . Biological controls on diurnal variations in seawater trace element concentrations and carbonate chemistry on a coral reef. Mar Chem 176, 1–8, 10.1016/j.marchem.2015.06.030 (2015).

[b30] Yan H. . Seasonal variations of seawater *p*CO_2_ and sea‐air CO_2_ fluxes in a fringing coral reef, northern South China Sea. J Geophys Res: Oceans 121, 998–1008, 10.1002/2015JC011484 (2016).

[b31] Lynch M. D. & Neufeld J. D. Ecology and exploration of the rare biosphere. Nat Rev Microbiol 13, 217–229, 10.1038/nrmicro3400 (2015). 25730701

[b32] Tout J. . Variability in microbial community composition and function between different niches within a coral reef. Microb Ecol 67, 540–552, 10.1007/s00248-013-0362-5 (2014). 24477921

[b33] Blackall L. L., Wilson B. & van Oppen M. J. Coral-the world’s most diverse symbiotic ecosystem. Mol Ecol 24, 5330–5347, 10.1111/mec.13400 (2015). 26414414

[b34] Haas A. F. . Global microbialization of coral reefs. Nat Microbiol 1, 16042, 10.1038/nmicrobiol.2016.42 (2016). 27572833

[b35] Tytlyanov E. A., Titlyanova T. V., Huang H. & Li X. Seasonal changes in benthic algal communities of the upper subtidal zone in Sanya Bay (Hainan Island, China). J Mar Biol Assoc UK 94, 51–64, 10.1017/S0025315413001112 (2014).

[b36] Ainsworth T. D. . The coral core microbiome identifies rare bacterial taxa as ubiquitous endosymbionts. ISME J 9, 2261–2274, 10.1038/ismej.2015.39 (2015). 25885563 PMC4579478

[b37] Bayer T. . The microbiome of the Red Sea coral *Stylophora pistillata* is dominated by tissue-associated *Endozoicomonas* bacteria. Appl Environ Microbiol 79, 4759–4762, 10.1128/AEM.00695-13 (2013). 23709513 PMC3719505

[b38] Wegley L., Edwards R., Rodriguez-Brito B., Liu H. & Rohwer F. Metagenomic analysis of the microbial community associated with the coral *Porites astreoides*. Environ Microbiol 9, 2707–2719, 10.1111/j.1462-2920.2007.01383.x (2007). 17922755

[b39] Krause E. . Small changes in pH have direct effects on marine bacterial community composition: a microcosm approach. PLoS One 7, e47035, 10.1371/journal.pone.0047035 (2012). 23071704 PMC3469576

[b40] Witt V., Wild C., Anthony K. R., Diaz-Pulido G. & Uthicke S. Effects of ocean acidification on microbial community composition of, and oxygen fluxes through, biofilms from the Great Barrier Reef. Environ Microbiol 13, 2976–2989, 10.1111/j.1462-2920.2011.02571.x (2011). 21906222

[b41] Webster N. S., Uthicke S., Botte E. S., Flores F. & Negri A. P. Ocean acidification reduces induction of coral settlement by crustose coralline algae. Glob Chang Biol 19, 303–315, 10.1111/gcb.12008 (2013). 23504741 PMC3597258

[b42] Loya Y. . Coral bleaching: the winners and the losers. Ecol Lett 4, 122–131, 10.1046/j.1461-0248.2001.00203.x (2001).

[b43] Hughes T. P. . Climate change, human impacts, and the resilience of coral reefs. Science 301, 929–933, 10.1126/science.1085046 (2003). 12920289

[b44] Howells E. J. . Coral thermal tolerance shaped by local adaptation of photosymbionts. Nat Clim Change 2, 116–120, 10.1038/nclimate1330 (2012).

[b45] Kelly L. W. . Local genomic adaptation of coral reef-associated microbiomes to gradients of natural variability and anthropogenic stressors. Proc Natl Acad Sci USA 111, 10227–10232, 10.1073/pnas.1403319111 (2014). 24982156 PMC4104888

[b46] Kroeker K. J. . Impacts of ocean acidification on marine organisms: quantifying sensitivities and interaction with warming. Glob Chang Biol 19, 1884–1896, 10.1111/gcb.12179 (2013). 23505245 PMC3664023

[b47] McConnaughey T. ^13^C and ^18^O isotopic disequilibrium in biological carbonates: I. Patterns. Geochim Cosmochim Ac 53, 151–162, 10.1016/0016-7037(89)90282-2 (1989).

[b48] Schoepf V. . Kinetic and metabolic isotope effects in coral skeletal carbon isotopes: A re-evaluation using experimental coral bleaching as a case study. Geochim Cosmochim Ac 146, 164–178, 10.1016/j.gca.2014.09.033 (2014).

[b49] Krief S. . Physiological and isotopic responses of scleractinian corals to ocean acidification. Geochim Cosmochim Ac 74, 4988–5001, 10.1016/j.gca.2010.05.023 (2010).

[b50] Carilli J. E., Charles C. D., Garren M., McField M. & Norris R. D. Baseline shifts in coral skeletal oxygen isotopic composition: a signature of symbiont shuffling? Coral Reefs 32, 559–571, 10.1007/s00338-012-1004-y (2013).

[b51] Brading P. . Differential effects of ocean acidification on growth and photosynthesis among phylotypes of *Symbiodinium* (Dinophyceae). Limnol Oceanogr 56, 927–938, 10.4319/lo.2011.56.3.0927 (2011).

[b52] Pierrot D., Lewis E. & Wallace D. MS excel program developed for CO_2_ system calculations. Carbon Dioxide Information Analysis Center, Oak Ridge National Laboratory, U.S. Department of Energy, Oak Ridge, Tennessee, (2006).

[b53] Cai L., Ye L., Tong A. H. Y., Lok S. & Zhang T. Biased diversity metrics revealed by bacterial 16S pyrotags derived from different primer sets. PLoS ONE 8, 10.1371/journal.pone.0053649 (2013). PMC3544912 23341963

[b54] Zhang J. J., Kobert K., Flouri T. & Stamatakis A. PEAR: a fast and accurate Illumina Paired-End read mergeR. Bioinformatics 30, 614–620, 10.1093/bioinformatics/btt593 (2014). 24142950 PMC3933873

[b55] Caporaso J. G. . QIIME allows analysis of high-throughput community sequencing data. Nat Methods 7, 335–336, 10.1038/nmeth.f.303 (2010). 20383131 PMC3156573

[b56] Edgar R. C. Search and clustering orders of magnitude faster than BLAST. Bioinformatics 26, 2460–2461, 10.1093/bioinformatics/btq461 (2010). 20709691

[b57] Wang Q., Garrity G. M., Tiedje J. M. & Cole J. R. Naive Bayesian classifier for rapid assignment of rRNA sequences into the new bacterial taxonomy. Appl Environ Microbiol 73, 5261–5267, 10.1128/AEM.00062-07 (2007). 17586664 PMC1950982

[b58] Pruesse E. . SILVA: a comprehensive online resource for quality checked and aligned ribosomal RNA sequence data compatible with ARB. Nucleic Acids Res 35, 7188–7196, 10.1093/nar/gkm864 (2007). 17947321 PMC2175337

