# Supplementary material for: Changes in microbial communities, photosynthesis and calcification of the coral Acropora gemmifera in response to ocean acidification
Source: Sci Rep. 2016 Oct 27;6:35971. doi: 10.1038/srep35971 (PMC5082368; doi:10.1038/srep35971)
Supplement: Supplementary Information [file srep35971-s1.pdf]

Supplementary information

Changes in microbial communities, photosynthesis and calcification of the coral *Acropora gemmifera* in response to ocean acidification

Guowei Zhou[1,2,3], Tao Yuan[1], Lin Cai[3], Weipeng Zhang[3], Renmao Tian[3], Haoya Tong[3], Lei Jiang[1], Xiangcheng Yuan[1], Sheng Liu[1], Peiyuan Qian[3*], Hui Huang[1,2*]

[1] Key Laboratory of Tropical Marine Bio-resources and Ecology, South China Sea Institute of Oceanology, Chinese Academy of Sciences, China

[2] Tropical Marine Biological Research Station in Hainan, Chinese Academy of Sciences, China

[3] Shenzhen Research Institute and Division of Life Science, Hong Kong University of Science and Technology, Hong Kong SAR, China

Running title: Responses of coral holobiont to ocean acidification

***Co-corresponding author:**

Peiyuan Qian, PhD

Shenzhen Research Institute and Division of Life Science, Hong Kong University of Science and Technology, Hong Kong SAR, China

Phone: +852-2358-7331
Fax: +852-2358-1559
E-mail: boqianpy@ust.hk

Hui Huang, PhD

South China Sea Institute of Oceanology, Chinese Academy of Sciences, 164 West Xingang Road, Guangzhou, China

Phone & Fax: +862-8446-0294

E-mail: huanghui@scsio.ac.cn

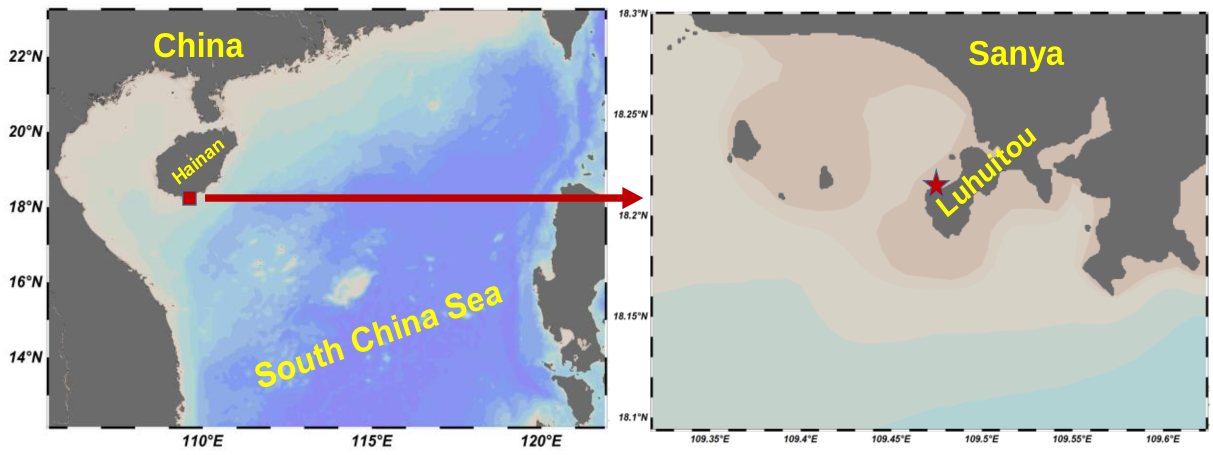

Figure S1. Maps of the coral *Acropora gemmifera* collecting site (shown in red star) on the Luhuitou fringing reef in southern Hainan Island, South China Sea. Base map was created using Ocean Data View (ODV 4.7.2, http://odv.awi.de).

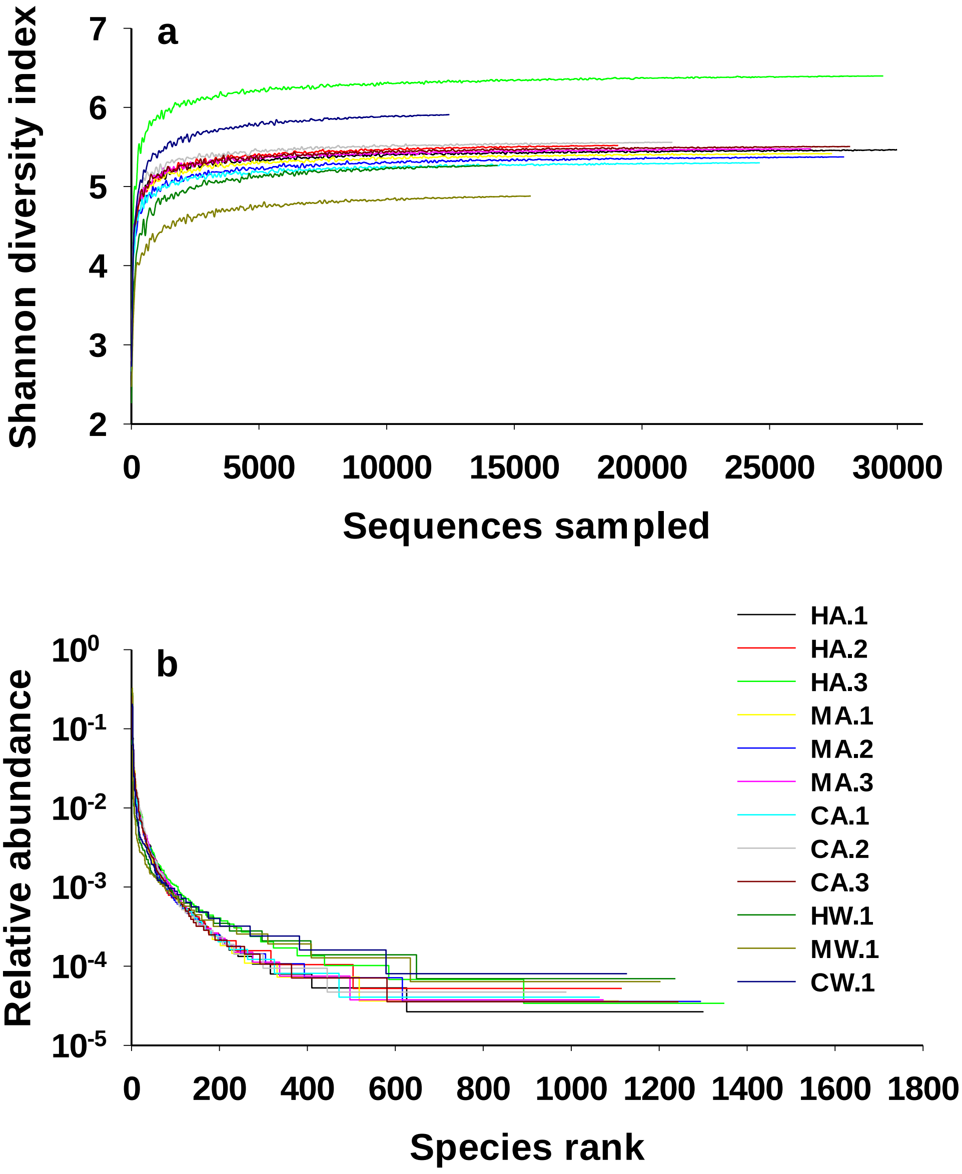

Figure S2. Rarefaction (a) and rank-abundance (b) curves of the 16S rRNA gene reads from each sample showing the diversity and abundance of the microbial communities in coral and seawater samples. The sample identifiers are shown in Table 2.

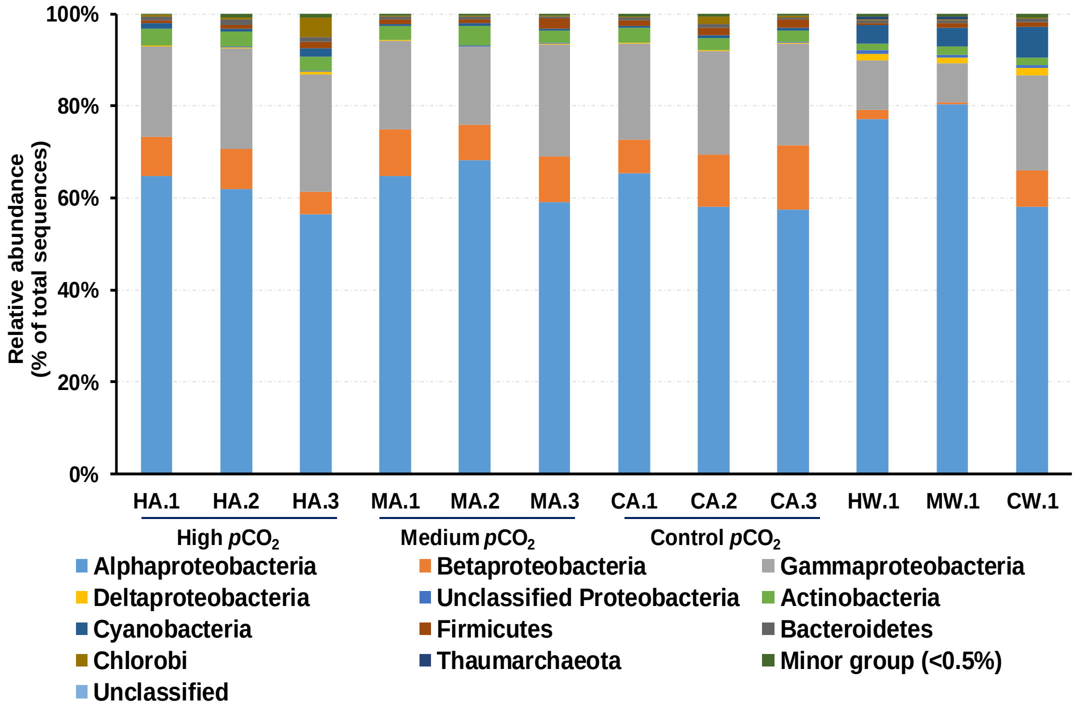

Figure S3. Coral and seawater microbial communities at the phylum level (except Proteobacteria shown at the class level). The minor group represents the sum of all phyla< 2% in all samples. For the sample identifiers, refer to Table 2.

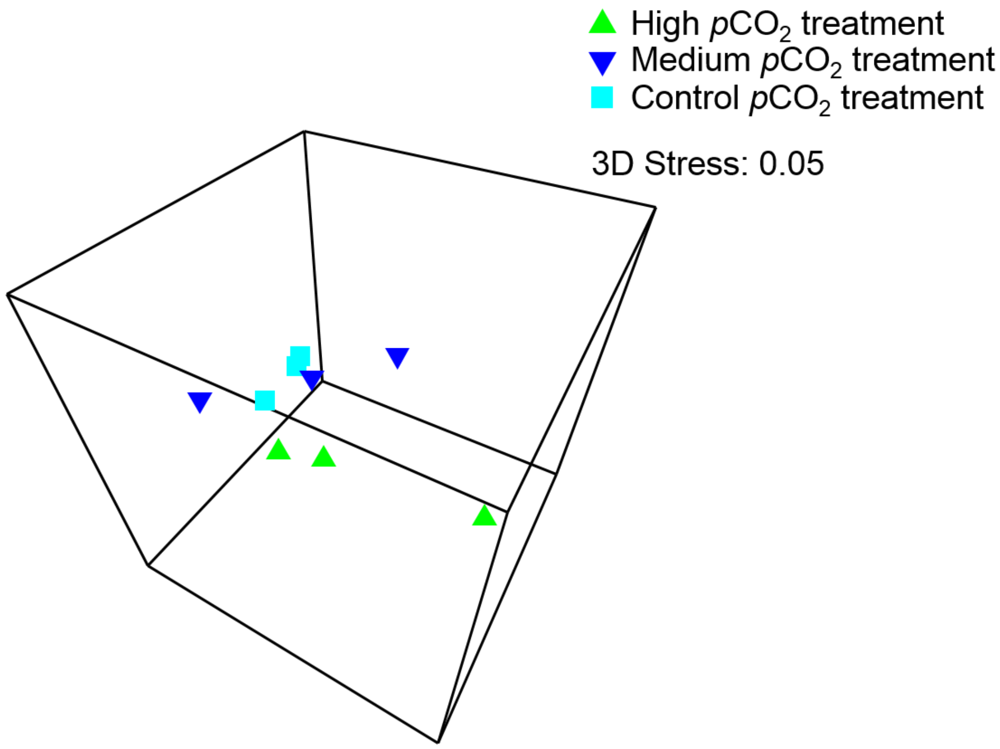

Figure S4. Non-metric multidimensional scaling (nMDS) 3-D plot of microbial communities among coral samples based on Bray–Curtis similarity indices.

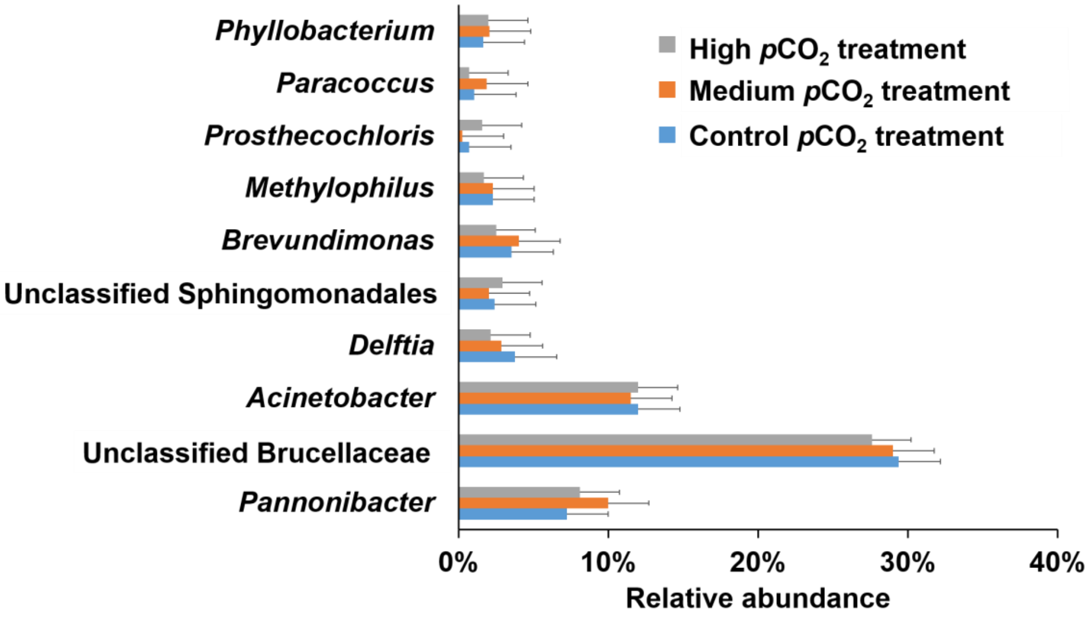

Figure S5. SIMPER analysis of the bacterial genera in coral samples under different *p*CO_2 treatments. SIMPER analysis were performed based on the Bray-Curtis distance using the relative abundance of bacterial genera as input. The top 10 genera that contribute to group difference are shown.

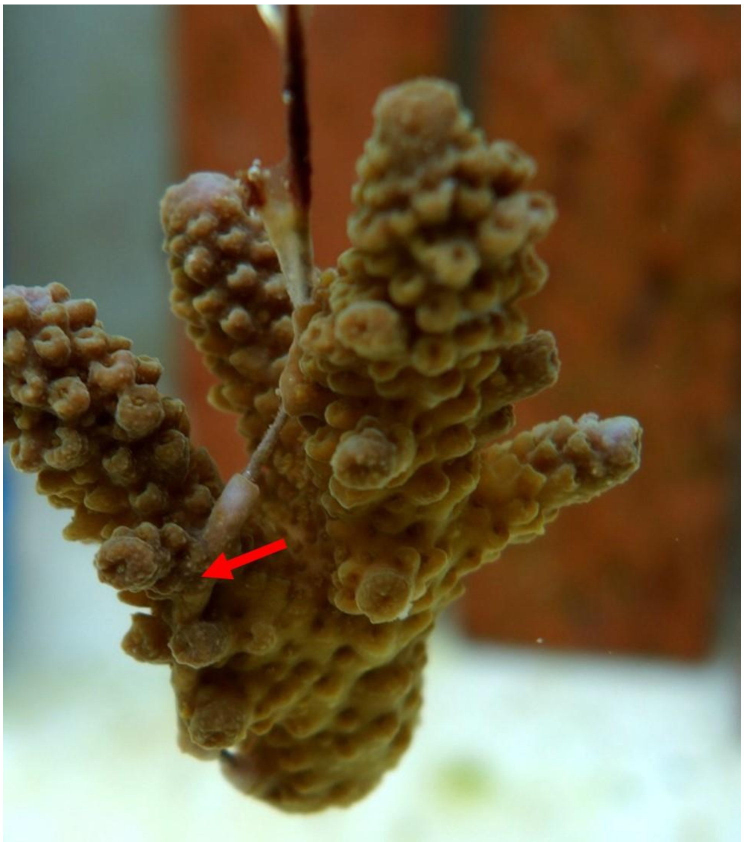

Figure S6. Picture of the coral *Acropora gemmifera* taken after 4-week exposure to the high pCO_2 treatment. The newly formatted skeleton was shown by red arrow.

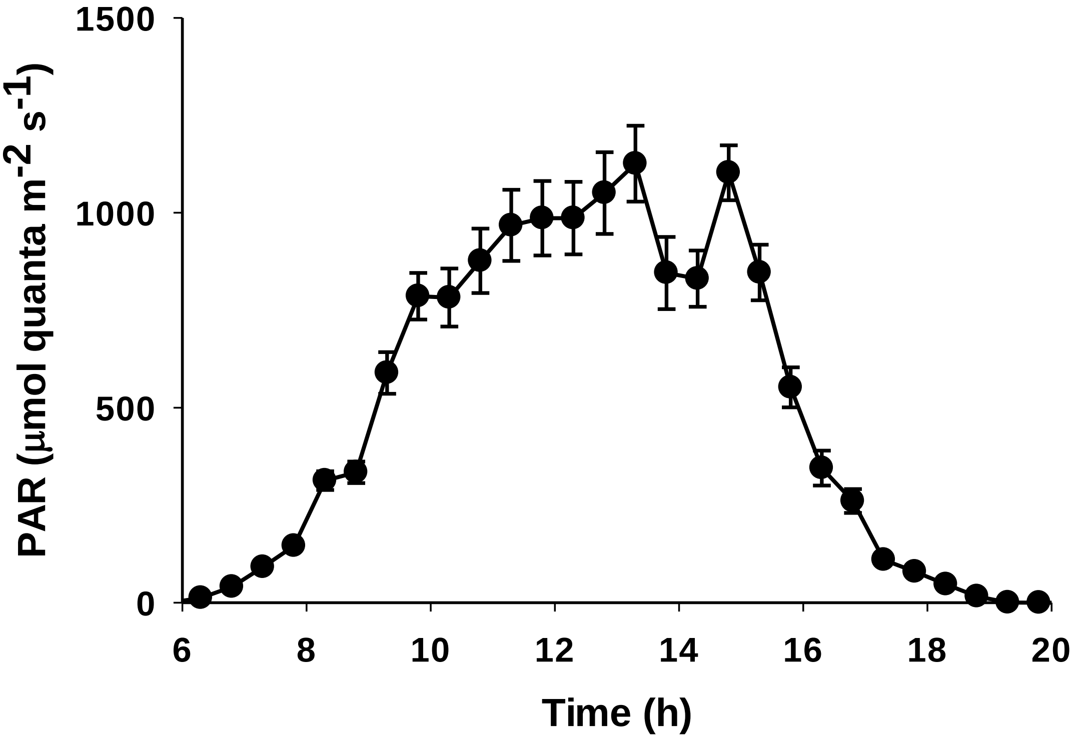

Figure S7. Average diurnal variations in photosynthetically active radiation (PAR) recorded in the tank during the 4-week treatments. Each point represents the average of the PAR record during the experiment with standard error bars.

Table S1. Summary of seawater pH and pCO_2 variation at the reef flat of Luhuitou fringing reef from published data (Ref.), if available. The numbers in red indicate the recorded extreme values.

Season	Observation Time	pH (range)	Seawater pCO_2 (range) µatm	Ref.
Spring	4/11/2010–4/14/2010	8.051 ±0.050 (7.864-8.221)	373 ±54 (231- 642)	1
	4/13/2011–4/24/2011	8.11 ±0.04	511 ±49 (377-679)	2
Summer	8/1/2009–8/7/2009	8.05±0.08	610 ±112 (360-**952**)	2
	6/28/2010–6/31/2010	8.007 ±0.048	420 ±62	1
	6/11/2011–6/18/2011	8.030 ±0.214 (**7.575**-8.379)	567 ±54 (189-**1542**)	3
Autumn	11/24/2010–12/3/2010	8.21 ±0.05	414 ±62 (233-623)	2
	11/19/2010–11/22/2010	8.035 ±0.077 (8.105 -8.375)	386 ±79	1
Winter	12/27/2009–12/30/2009	8.016 ±0.033 (8.048- 8.248)	408 ±40 (225-387)	1
	2/19/2011–3/1/2011	8.15±0.05	428 ±48 (329-623)	2

References:

1 Zhang, C. L. *et al.* Diurnal and seasonal variations of carbonate system parameters on Luhuitou fringing reef, Sanya Bay, Hainan Island, South China Sea. *Deep-Sea Res Pt Ii* **96**, 65-74, doi:10.1016/j.dsr2.2013.02.013 (2013).

2 Yan, H. *et al.* Seasonal variations of seawater pCO_2 and sea−air CO_2 fluxes in a fringing coral reef, northern South China Sea. *J Geophys Res: Oceans* **121**, 998–1008, doi: 10.1002/2015JC011484 (2016).

3 Chen, X. F. *et al.* Biological controls on diurnal variations in seawater trace element concentrations and carbonate chemistry on a coral reef. *Mar Chem* **176**, 1-8, doi:10.1016/j.marchem.2015.06.030 (2015).
